# Ionic liquid-enhanced recycling of lithium-ion battery black mass *via* heavy liquid centrifugal separation

**DOI:** 10.1039/d5ra07158e

**Published:** 2026-01-02

**Authors:** Babafemi Adigun, Huimin Luo, Tao Wang, Sheng Dai

**Affiliations:** a Department of Chemistry, Institute for Advanced Materials and Manufacturing, University of Tennessee Knoxville Tennessee 37996 USA; b Manufacturing Science Division, Oak Ridge National Laboratory Oak Ridge Tennessee 37831 USA; c Chemical Sciences Division, Oak Ridge National Laboratory Oak Ridge Tennessee 37831 USA wangt@ornl.gov dais@ornl.gov

## Abstract

Direct recycling of lithium-ion batteries (LIBs) is of great significance to supply chain security and environmental protection by restoring spent battery materials to their original purpose without destroying their chemical structure. One of the key processes for direct recycling is to separate valuable anode and cathode active materials from black mass. This study evaluates ionic liquid-enhanced Heavy Liquid Centrifugal Separation (HLCS) as an efficient method for separating LIB black mass into its constituent anode and cathode materials. The optimized HLCS process achieved a separation efficiency of over 95%, yielding a graphite-rich upper layer and an NMC-rich lower layer. Characterization by thermogravimetric analysis (TGA), X-ray diffraction (XRD), and inductively coupled plasma-optical emission spectroscopy (ICP-OES) confirmed the purity and structural integrity of the recovered fractions. The addition of *N*-methyl-2-pyrrolidone and ionic liquid 1-ethyl-3-methylimidazolium bromide decoupled entangled particles, while subsequent treatment of the anode layer with 1-(2,3-dihydroxypropyl)-3-methylimidazolium chloride further enhanced separation purity. The recycled graphite exhibited comparable battery performance to pristine graphite. These results demonstrate HLCS as a promising LIB recycling strategy, advancing sustainable battery manufacturing.

## Abstract 2

Direct recycling of lithium-ion batteries (LIBs) is of great significance to supply chain security and environmental protection by restoring spent battery materials to their original purpose without destroying their chemical structure.

## Graphical abstract

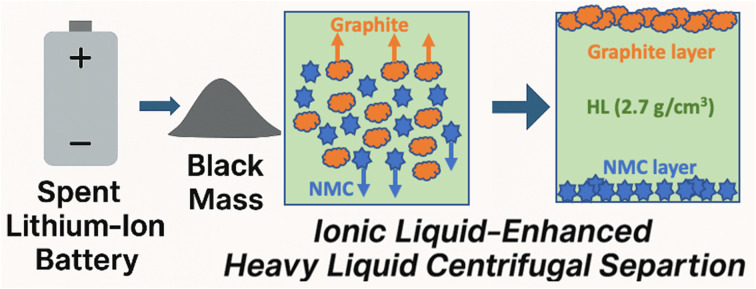

## Introduction

Lithium-ion batteries (LIBs) are at the forefront of the energy transition towards more sustainable power sources powering everything from portable electronics to electric vehicles (EVs). Their high energy density, long lifespan, and versatility make them a preferred choice for a wide range of applications.^1–5^ The rapid expansion of the electric vehicle (EV) market, along with the widespread use of LIBs in portable electronics, has precipitated unprecedented growth in the LIB industry. However, the end-of-life management of these batteries presents significant environmental challenges and opportunities for resource recovery.^6,7^ Lithium battery recycling emerges as a critical solution to mitigate these environmental impacts, offering a pathway to recover valuable materials, such as lithium, cobalt, and nickel, thereby diminishing the demand for resource extraction and reducing the overall environmental burden.^8,9^ Beyond environmental considerations, the strategic importance of lithium battery recycling extends to enhancing material sustainability and economic resilience. Many of the raw materials integral to LIBs are sourced from geopolitically sensitive regions, subjecting supply chains to potential disruptions. By establishing robust recycling capabilities, we can alleviate dependence on uncertain raw material supplies, fostering a more stable and sustainable battery supply chain.^10,11^

Lithium battery recycling is currently dominated by three primary technologies: pyrometallurgy, hydrometallurgy, and direct recycling. Pyrometallurgical processes, involving high-temperature smelting, are favored for their operational robustness and ability to recover valuable metals such as cobalt, nickel, and copper. However, this method is energy intensive.^12–17^ Hydrometallurgical processes offer a more environmentally benign alternative, using aqueous solutions to leach metals from battery waste. This method allows for the selective recovery of metals, including lithium, though it requires precise chemical management and can produce hazardous wastewater if not properly controlled.^18–20^ Direct recycling stands out for its innovative approach and distinguishes itself from conventional recycling methods by aiming to preserve the structural and chemical integrity of cathode and anode materials for reuse in new batteries. The process involves several key steps: disassembly of spent batteries, separation and purification of electrode materials, and their direct reintroduction into new battery manufacturing. Unlike pyrometallurgical and hydrometallurgical processes, which primarily recover metal constituents through smelting and chemical leaching, direct recycling targets the preservation of valuable electrochemical properties of the active materials.^21,22^ Despite these advantages of direct recycling, the handling of black mass remains a bottleneck for scalable direct recycling systems. During shredding, graphite, carbon black, and cathode active material often form dense agglomerates due to strong PVDF binding, leading to poor particle separation, contamination, and reduced phase purity during separation steps. Many physical separation technologies such as froth flotation,^27,28^ magnetic separation^29–31^ are highly sensitive to surface chemistry, particle size distribution, and residual binder content. This complexity drives the need for hybrid approaches that combine physical separation with targeted chemical assistance to enhance selectivity and yield (Table 1).

**Table 1:** Quick comparison table of ILs for separation and purification

Ionic liquid	Method	Application	Ref.
1-Decyl 3-methylimidazolium bis (trifluoromethylsulfonyl) imide	Solvent extraction	Diluent	23
1-Butyl-3-methylimidazolium tetrafluoroborate ([BMIm][BF_4_])	Binder dissolution	PVDF binder removal	24
1-(2,3-Dihydroxypropyl)-3-methylimidazolium chloride ([DHPMim][Cl])	Metal dissolution	Selective metal-oxide dissolution	25
Tri(hexyl)tetradecyl phosphonium chloride [P_66614_][Cl]	Supported IL membrane	Extractant	26

In the process of direct recycling, a physical separation approach is employed to disassemble the battery into its constituent parts. This process generally starts with the mechanical destruction of batteries through methods like shredding or crushing. This step is followed by a classification procedure that segregates components based on their size. The larger fragments, which include plastics and current collectors, are isolated from the finer materials known as the black mass. The black mass is particularly significant as it comprises the active material from both the anode and cathode, including graphite, polyvinylidene fluoride (PVDF), carbon black, and lithium transition metal oxides, in addition to traces of aluminum and copper from the current collectors.^32–34^ Various methodologies aimed at improving the efficiency and sustainability of recycling processes. One such method, gravity separation, has emerged as a potentially viable technique for the separation of black mass components in LIBs. Heavy liquid centrifugal separation (HLCS) is a method that leverages the differences in density between the various components of the black mass to achieve separation. Black mass, the residue obtained after the mechanical processing of spent LIBs, contains a mixture of cathode and anode materials, including valuable metals such as lithium, cobalt, nickel, and manganese, as well as graphite. The principle of HLCS involves the use of fluid dynamics and the differential settling rates of particles to segregate materials based on their density.^35,36^ Heavy liquid (HL) separation techniques have been utilized to separate anode and cathode materials. Specifically, cathode active materials such as lithium cobalt oxide (LCO) and lithium nickel manganese cobalt oxide (NMC) have densities ranging from 4.5 to 5.0 g cm^−3^, significantly higher than the density of graphite, which is 2.26 g cm^−3^. By employing heavy liquids with densities between 2.26–4.50 g cm^−3^, it is possible to achieve effective separation of these two types of materials when mixed.^25,37,38^ Al-Shammari *et al.* utilized sodium polytungstate (SPT) as a heavy liquid for the recovery of graphite from mixed spent LIB powders. The recovered graphite delivered a specific capacity that is nearly identical to that of commercial graphite, demonstrating its excellent electrochemical performance and retention of functional integrity.^39^ Their practical efficiency was constrained by particle entanglement, incomplete binder removal, and sluggish settling dynamics. These limitations underscore the need for intelligent modification of the liquid medium to promote dispersion and minimize interparticle adhesion.^40^

Ionic liquids (ILs) have rapidly emerged as versatile functional materials in battery recycling due to their ability to disrupt PVDF adhesion, leaching of metal ions and act as selective extractants for recovering metal ions from spent batteries. Recent studies have demonstrated that ILs can dissolve PVDF binder, enabling easier detachment of cathode particles. Zeng *et al.* demonstrated that the ionic liquid 1-butyl-3-methylimidazolium tetrafluoroborate ([BMIm][BF_4_]), when heated to 180 °C under continuous stirring (300 rpm), effectively cleaved the PVDF binder.^24,41^ ILs have also been used as extractants for cobalt and lithium from spent batteries.^26,42–44^ Wang *et al.* employed an ionic liquid system composed of 1-butyl-3-methylimidazolium bis(trifluoromethylsulfonyl)imide ([BMIm][NTf_2_]), 2-thenoyltrifluoroacetone (HTTA), and trioctylphosphine oxide (TOPO) to efficiently extract and recover valuable transition metals from spent lithium-ion batteries.^45^ Their multipurpose functionality, tunable physicochemical properties, and chemical stability under battery-recycling conditions position ILs as effective enhancers that can improve physical separation processes without degrading the active materials.

In this study, we investigate the direct recycling of lithium-ion black mass using ionic liquid enhanced heavy liquid centrifugal separation to selectively recover anode and cathode active materials from black mass. Bromoform, a high-density organic liquid with an intermediate density between the cathode and anode materials, was employed as the dense medium to facilitate the separation. However, the separation performance of pure bromoform is limited by the complex composition and entangled particles in black mass. By choosing ionic liquids and NMP as additives, the formulated heavy liquids exhibited excellent performance for black mass separation, generating NMC and graphite products with high purities of over 95%. The recycled graphite was treated with IL to remove residual cathode materials from the graphite, enabling electrochemical performance comparable to pristine anode material. These emerging synergies between HLCS and ionic-liquid chemistry provide a promising route to enhance the selectivity and recovery of high-value components during direct recycling.

## Material and methods

### Materials

The spent black mass used in this study was provided by a commercial vendor, which was mechanically shredded and sieved from lithium-ion battery waste composed of graphite as the anode material and lithium nickel-cobalt-manganese oxide (NMC) as the cathode material. *N*-Methyl-2-pyrrolidone (NMP, ≥99%, Sigma-Aldrich), bromoform (96% Aldrich Chemical Company), 1-ethyl-3-methylimidazolium bromide (C_2_mimBr, 98%, Thermo Scientific), 1-(2,3-dihydroxypropyl)-3-methylimidazolium chloride prepared as synthesized in literature.^25^

### Methods

#### Separation of anode and cathode layer

The black mass was first ground using a mortar and pestle until no visible lumps remained. The resulting material was then sieved through a 150 µm mesh to obtain a fine, uniform powder suitable for separation. A heavy liquid mixture was prepared using bromoform (BF) and/or a combination of bromoform, IL, and NMP. The black mass and liquid mixture were combined at a mass-to-volume ratio of 1 : 4 (corresponding to 2 g black mass, 360 µL NMP, 1 mL ionic liquid, and 7 mL bromoform). The mixture was sonicated for 20 minutes to ensure uniform particle dispersion, followed by stirring for 5 hours to enhance the interaction between the heavy liquid and the electrode particles. After treatment, the mixture was centrifuged at 5000 rpm for 3 minutes, resulting in clear separation of two solid layers due to the density difference between the cathode and anode materials. The bottom layer (cathode-rich fraction) and the upper layer (graphite-rich fraction) were carefully collected using a separatory funnel. Both layers were rinsed with acetone and deionized water to remove any residual bromoform and ionic liquid, then dried under vacuum overnight at 70 °C. The dried products were subsequently used for thermogravimetric analysis (TGA), X-ray diffraction (XRD), and electrochemical testing.

### Characterization

The half-cell tests were performed using lithium foil disks as counter and reference electrodes. The anode slurry, containing pristine or recycled graphite, carbon black, and PVDF, in a mass ratio of 92 : 2 : 6 with NMP, was coated onto copper foil. Coin cells were assembled in an argon-filled glovebox using Celgard 2320 membrane as the separator. The electrolyte is 1.2 M LiPF_6_ in 30 : 70 wt% ethylene carbonate/ethyl methyl carbonate.

The composition of the separated layers was determined using thermogravimetric analysis (TGA), where the recovered anode and cathode layers from the separation were heated above the thermal decomposition temperature of the anode material at a scanning rate of 10 °C min^−1^ in the temperature range of 25–900 °C under an air atmosphere. The residual mass percentage of the sample at 900 °C corresponds to the mass fraction of the cathode, whereas the weight loss percentage represents the fraction of graphite present in each layer. The chemical composition and purity of the recovered electrode materials were analyzed using inductively coupled plasma-optical emission spectroscopy (ICP-OES) and X-ray diffraction (XRD).

## Results and discussion

### Separation of cathode/anode mixture

**Fig. 1:**
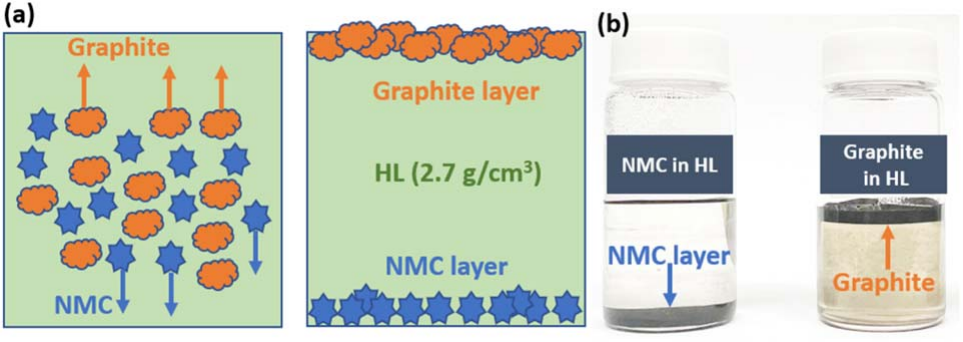
(a) Illustration of NMC/Graphite separation in HL. (b) Photos of NMC and graphite in HL.

**Fig. 2:**
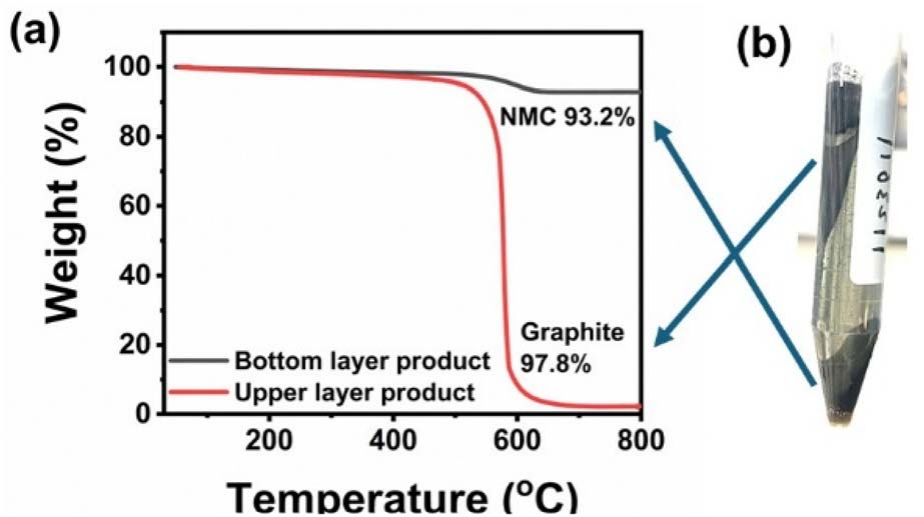
(a) TGA curves of the separated bottom and upper layers. (b) Photo of graphite/NMC mixtures after centrifugal separation.

The mechanical separation of mineral or electrode particles in a dense liquid can be qualitatively explained using Stokes' law, which describes the motion of small, near-spherical particles settling or floating due to density differences between the particle and the surrounding medium. In such systems, particle movement is governed by the balance of gravitational, buoyant, and drag forces, and under ideal conditions the separation behavior can be reliably predicted. This framework forms the basis for understanding how black-mass particles behave in heavy-liquid environments and why density contrast plays a critical role in determining their separation efficiency.^36,39^ Selective separation of the anode and cathode fractions was achieved using a heavy liquid whose density was carefully chosen to lie between that of the cathode and the anode active materials. The cathode and anode active materials have densities of approximately 4.7 g cm^−3^ and 2.3 g cm^−3^, respectively. For effective separation, bromoform/ionic liquid/NMP solution, with a density of 2.7 g cm^−3^, was chosen as the heavy liquid, falling within the density range between the cathode and anode active materials. A mixture of pristine graphite anode and NMC cathode materials was prepared for this study. Equal masses (1 g each) of NMC cathode and graphite anode were combined with bromoform as a heavy liquid. The mixture was sonicated for 20 minutes and subsequently stirred for 5 hours to ensure thorough dispersion. It was then subjected to centrifugation at 5000 rpm for 3 minutes. Due to differences in specific densities, the graphite floated to the surface while the NMC cathode settled at the bottom. This separation process enables the complete recovery of the anode active material from the cathode using a separatory funnel. Fig. 1a illustrates the separation of a cathode/anode mixture using a heavy liquid. Fig. 1b shows the distribution of the cathode and anode within the heavy liquid, highlighting their displacement as a function of density differences. The separation performance was evaluated through TGA and XRD of the separated electrode materials, namely graphite (anode layer) and NMC cathode (cathode layer). Fig. 2a presents the TGA analysis of the cathode/anode mixture following its separation into distinct layers. The bottom layer, primarily composed of cathode material, exhibits a 6.8% weight loss upon heating beyond the thermal decomposition temperature of the anode material, indicating that cathode active material constitutes approximately 93.2% of this layer. The upper layer, predominantly consisting of anode material, undergoes a 97.8% weight loss, corresponding to the proportion of anode active material. A control experiment using pure bromoform for the separation of NMC/graphite exhibited much lower purity for graphite (85%) and NMC (79%) products (Fig. S1(a)), which highlights the advantages of ionic liquid and NMP additives in improving the separation performance of heavy liquids.

To further analyze the composition of the cathode/anode mixture before and after separation, X-ray diffraction (XRD) was performed on both the mixed and separated layers. Fig. S1(b and c) presents the XRD patterns of pristine graphite and the NMC cathode, with characteristic diffraction peaks observed at 26.4° and 18.8°, respectively, confirming the presence of these phases. Following the separation of the cathode/anode mixture in bromoform, Fig. S1(d and e) display the XRD patterns of the upper layer (anode layer) and the bottom layer (NMC cathode layer). The XRD pattern of the separated anode layer (Fig. S1(d)) predominantly exhibits the graphite peak, along with a minor peak indicating residual NMC cathode. Similarly, the XRD pattern of the separated cathode layer (Fig. S1(e)) reveals major peaks of the NMC cathode and graphite, aligning with the TGA data presented in Fig. S1(a). These results suggest that the separation process does not achieve complete phase purity, as some cathode material remains in the anode layer and *vice versa*. To enhance the efficiency of the separation, *N*-methyl-2-pyrrolidone (NMP) and the ionic liquid 1-ethyl-3-methylimidazolium bromide (C_2_mimBr) were introduced in conjunction with the heavy liquid. These additives can help dissolve active materials typically bound by polymeric binders such as polyvinylidene fluoride (PVDF) and facilitate the dispersion of solid particles in the liquid phase, preventing agglomeration. Fig. S2 presents the XRD patterns of the separated anode and cathode layers after incorporating additives (NMP, C_2_mimBr, and a combination of both) into the heavy liquid. The objective was to evaluate the impact of these additives on improving phase separation. The XRD patterns of the anode layer (Fig. S2(a–c)) suggest that the addition of NMP, C_2_mimBr, or their combination had minimal impact on improving the separation of anode material, as no significant enhancement was observed compared to using the heavy liquid alone. Peaks corresponding to residual cathode material remain present, suggesting that the additives had a limited effect on further purifying the anode layer. The cathode layer exhibited a marked improvement in separation efficiency upon the addition of the additives. Compared to using the heavy liquid alone, the incorporation of NMP and C_2_mimBr led to a substantial reduction in the intensity of peaks corresponding to anode material in the cathode layer (Fig. S2(d and e)). Among the tested conditions, the combined use of NMP and C_2_mimBr resulted in the most effective separation (Fig. S2(f)), indicating their synergistic role in enhancing the removal of residual anode material from the cathode fraction.

### Separation of black mass

**Fig. 3:**
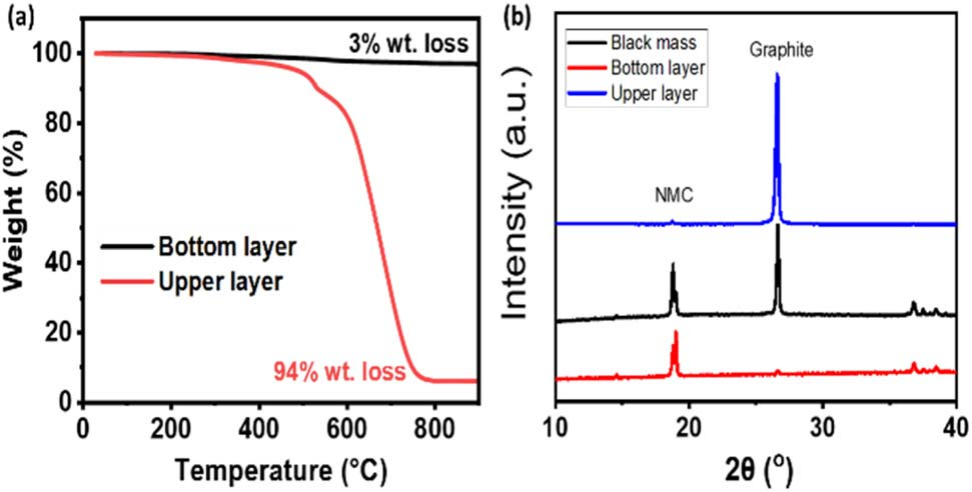
(a) TGA curves of bottom and upper layer products from black mass. (b) XRD patterns of black mass and bottom/upper layer products.

The composition of the NMC cathode and metal content in the black mass was determined using inductively coupled plasma-optical emission spectroscopy (ICP-OES), is presented in Table S1. Based on these results, the NMC cathode in the black mass was calculated to have a composition of LiNi_0_._39_Mn_0_._27_Co_0_._38_O_2_. To evaluate the effectiveness of the separation process under real-world conditions, black mass from spent batteries was subjected to cathode/anode separation using a combination of NMP and C_2_mimBr as additives in the heavy liquid. 10 g of black mass was separated into cathode and anode active materials. The TGA of the separated layers is shown in Fig. 3a. The cathode layer exhibited only a 3% weight loss upon heating beyond the thermal decomposition temperature of the anode material, indicating that it consisted of approximately 97% cathode active material. Conversely, the anode layer demonstrated a 94% weight loss, confirming a 94% composition of anode-active material. Further characterization using X-ray diffraction (XRD) provided additional insights into the separation efficiency. Fig. 3b presents the XRD pattern of the black mass before separation, displaying characteristic peaks for both graphite and the NMC cathode at nearly equal intensities. After separation, the XRD pattern of the anode layer predominantly exhibited the graphite peak, with only a minor peak corresponding to the NMC cathode, indicating minimal cathode contamination. Similarly, the XRD pattern of the cathode layer revealed strong peaks for the NMC cathode, with either a negligible or nearly undetectable graphite peak, signifying the effective removal of anode material from the cathode fraction. The separation process achieved an 85% recovery of the total electrode active materials. Of this, 28% corresponded to anode material (predominantly graphite), while 57% consisted of cathode active material (NMC), as detailed in Table S2. The total recovery rate (85%) was determined using the actual mass of black mass (10 g) introduced into the HLCS process. The sum of the recovered anode and cathode fractions correspond to 8.5 g, equivalent to 85% of the total mass input. These results demonstrate that the formulated Bromoform/NMP/C_2_mimBr heavy liquid significantly enhances cathode/anode separation, leading to a high-purity recovery of both active materials.

**Fig. 4:**
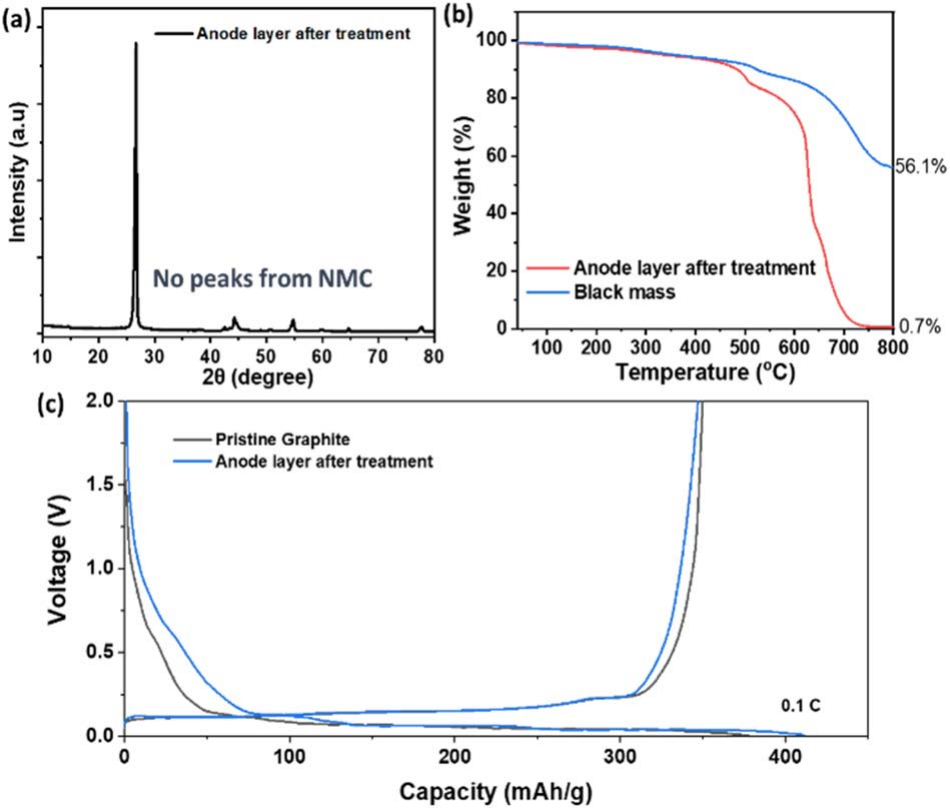
(a) XRD patterns of anode layer from black mass after treatment with ionic liquids. (b) TGA curves of black mass and anode layer products from black mass after treatment with ionic liquids. (c) The first charge/discharge curves of graphite|Li.

To further improve the purity of the anode layer and eliminate residual cathode material, the separated anode fraction underwent additional treatment with 1-(2,3-dihydroxypropyl)-3-methylimidazolium chloride, an ionic liquid (IL) known for its effectiveness in dissolving metal oxides.^25^ The IL was introduced to the anode layer at a mass ratio of 5 : 1 (IL to anode material), and the mixture was subjected to continuous stirring at 120 °C for 12 hours. Following the treatment, the solid fraction was thoroughly washed with ethanol and deionized water to remove any residual IL and impurities. The effectiveness of this purification step was evaluated using X-ray diffraction (XRD). As shown in Fig. 4a, the XRD pattern of the anode layer after IL treatment revealed the complete disappearance of cathode-related peaks, confirming the successful removal of residual cathode material. The TGA analysis of the anode exhibits more than 99% weight loss corresponding to the percentage of graphite in the anode layer further confirming the removal of the cathode materials from the anode layer (Fig. 4b). The recovered graphite after the purification step with IL was used as anode active materials for graphite|Li half-cell battery tests. The recovered graphite exhibited almost identical charge/discharge curves to pristine graphite (Fig. 4c). The ionic liquid purification step effectively eliminates cross-contamination between graphite and NMC layers, reducing cathode residues in the anode fraction to <1 wt%. The recycled graphite shows identical charge/discharge behavior to pristine graphite, indicating no internal short-circuit risk.

## Conclusions

This study highlights the potential of ionic liquid-enhanced Density Liquid Centrifugal Separation (HLCS) using bromoform for direct recycling of lithium-ion battery (LIB) black mass. The method effectively exploits density differences to achieve selective separation of graphite from NMC cathode materials achieving high-purity fractions. The use of ionic liquid C_2_mimBr and NMP as additives improve cathode separation but has minimal effect on anode purification, implying that factors like particle wettability and surface interactions influence the process efficiency. Further treatment with ionic liquid 1-(2,3-dihydroxypropyl)-3-methylimidazolium chloride effectively removes residual cathode material from the separated anode layer. These results highlight the potential of HLCS as an efficient method for the direct recycling of black mass.

## Author contributions

CRediT: Babafemi Adigun: writing – original draft, investigation; Tao Wang: supervision, writing – review & editing, methodology; Huimin Luo: writing – review & editing, funding acquisition; Sheng Dai; writing – review & editing, funding acquisition, conceptualization.

## Conflicts of interest

There are no conflicts to declare.

## Supplementary Material

RA-016-D5RA07158E-s001

## Data Availability

Supporting data for this study are available in the supplementary information (SI). Supplementary information: metal composition of black mass, yield and composition of separated electrode active materials from black mass, TGA curves of the separated layers, XRD pattern for the separated layers, average crystal sizes of NMC cathode and graphite, half-cell cycling performance of anode layer after treatment. See DOI: https://doi.org/10.1039/d5ra07158e.

## References

[cit1] Nitta N., Wu F., Lee J. T., Yushin G. (2015). Mater. Today.

[cit2] Folayan T.-O., Lipson A. L., Durham J. L., Pinegar H., Liu D., Pan L. (2021). Energy Technol..

[cit3] Manthiram A. (2020). Nat. Commun..

[cit4] Zhu X.-N., Nie C.-C., Wang S.-S., Xie Y., Zhang H., Lyu X.-J., Qiu J., Li L. (2020). J. Clean. Prod..

[cit5] Li W., Erickson E. M., Manthiram A. (2020). Nat. Energy.

[cit6] Duarte Castro F., Vaccari M., Cutaia L. (2022). Crit. Rev. Environ. Sci. Technol..

[cit7] Mossali E., Picone N., Gentilini L., Rodrìguez O., Pérez J. M., Colledani M. (2020). J. Environ. Manag..

[cit8] Zhang W., Xu C., He W., Li G., Huang J. (2018). Waste Manag. Res..

[cit9] Chen M., Ma X., Chen B., Arsenault R., Karlson P., Simon N., Wang Y. (2019). Joule.

[cit10] Liu W., Li X., Liu C., Wang M., Liu L. (2023). Resour. Policy.

[cit11] Hira A. (2025). Int. J..

[cit12] Zhang X., Li L., Fan E., Xue Q., Bian Y., Wu F., Chen R. (2018). Chem. Soc. Rev..

[cit13] Huang B., Pan Z., Su X., An L. (2018). J. Power Sources.

[cit14] Lv W., Wang Z., Cao H., Sun Y., Zhang Y., Sun Z. (2018). ACS Sustain. Chem. Eng..

[cit15] Harper G., Sommerville R., Kendrick E., Driscoll L., Slater P., Stolkin R., Walton A., Christensen P., Heidrich O., Lambert S. (2019). Nature.

[cit16] Makuza B., Tian Q., Guo X., Chattopadhyay K., Yu D. (2021). J. Power Sources.

[cit17] Zhou M., Li B., Li J., Xu Z. (2021). ACS ES&T Eng..

[cit18] Asadi Dalini E., Karimi G., Zandevakili S., Goodarzi M. (2021). Miner. Process. Extr. Metall. Rev..

[cit19] Chagnes A., Pospiech B. (2013). J. Chem. Technol. Biotechnol..

[cit20] Yao Y., Zhu M., Zhao Z., Tong B., Fan Y., Hua Z. (2018). ACS Sustain. Chem. Eng..

[cit21] Ji H., Wang J., Ma J., Cheng H.-M., Zhou G. (2023). Chem. Soc. Rev..

[cit22] Sloop S., Crandon L., Allen M., Koetje K., Reed L., Gaines L., Sirisaksoontorn W., Lerner M. (2020). Sustain. Mater. Technol..

[cit23] Zante G., Masmoudi A., Barillon R., Trébouet D., Boltoeva M. (2020). J. Ind. Eng. Chem..

[cit24] Zeng X., Li J. (2014). J. Hazard. Mater..

[cit25] Hu Y., Yang M., Dong Q., Zou X., Yu J., Guo S., Yan F. (2024). Energy Environ. Sci..

[cit26] Zante G., Boltoeva M., Masmoudi A., Barillon R., Trébouet D. (2020). Sep. Purif. Technol..

[cit27] Vanderbruggen A., Hayagan N., Bachmann K., Ferreira A., Werner D., Horn D., Peuker U., Serna-Guerrero R., Rudolph M. (2022). ACS ES&T Eng..

[cit28] Verdugo L., Zhang L., Etschmann B., Bruckard W., Menacho J., Hoadley A. (2023). Sep. Purif. Technol..

[cit29] Mennik F., Dinç N. İ., Burat F. (2023). Results Eng..

[cit30] Hu Z., Liu J., Gan T., Lu D., Wang Y., Zheng X. (2022). Sep. Purif. Technol..

[cit31] Ding W., Bao S., Zhang Y., Xin C., Chen B., Li J., Liu B., Xia Y., Hou X., Xu K. (2024). J. Clean. Prod..

[cit32] Xu P., Tan D. H., Jiao B., Gao H., Yu X., Chen Z. (2023). Adv. Funct. Mater..

[cit33] Lu Y., Peng K., Zhang L. (2022). ACS ES&T Eng..

[cit34] Wang H., Burke S., Yuan R., Whitacre J. F. (2023). J. Energy Storage.

[cit35] Zhang Y., He Y., Zhang T., Zhu X., Feng Y., Zhang G., Bai X. (2018). J. Clean. Prod..

[cit36] Al-Shammari H., Farhad S. (2021). Resour. Conserv. Recycl..

[cit37] Zhan R., Pan L. (2022). Sustainable Mater. Technol..

[cit38] Kepler K.D. , Tsang F., Vermeulen R. and Hailey P., US Pat., 9614261B2, 2017

[cit39] Al-Shammari H., Alharbi S., Bashir M. B. A. (2025). Int. J. Energy Res..

[cit40] Roy J. J., Phuong D. M., Verma V., Chaudhary R., Carboni M., Meyer D., Cao B., Srinivasan M. (2024). Carbon Energy.

[cit41] Li A., Sun Z., Lv C., Fu Z. (2025). Sep. Purif. Technol..

[cit42] Meshram P., Agarwal N. (2025). RSC Adv..

[cit43] Morina R., Merli D., Mustarelli P., Ferrara C. (2023). Chemelectrochem.

[cit44] Xu L., Chen C., Fu M.-L. (2020). Hydrometallurgy.

[cit45] Wang K., Zhang G., Luo M., Li J. (2023). Chem. Eng. J..

