# Supplementary material for: Ionic liquid-enhanced recycling of lithium-ion battery black mass via heavy liquid centrifugal separation
Source: RSC Adv. 2026 Jan 2;16(1):568–73. doi: 10.1039/d5ra07158e (PMC12757933; doi:10.1039/d5ra07158e)
Supplement: RA-016-D5RA07158E-s001 [file RA-016-D5RA07158E-s001.pdf]

Supplementary Information (SI) for RSC Advances.
This journal is © The Royal Society of Chemistry 2025

Ionic Liquid-Enhanced Recycling of Lithium-Ion Battery Black Mass via Heavy Liquid Centrifugal Separation

Babafemi Adigun,[a] Huimin Luo,[b] Tao Wang,*[c] Sheng Dai*[a,c]

[a] Department of Chemistry, Institute of Advanced Materials & Manufacturing, The University of Tennessee, Knoxville, Tennessee 37996, United States.

[b] Manufacturing Science Division, Oak Ridge National Laboratory, Oak Ridge, TN, 37831, United States.

[c] Chemical Sciences Division, Oak Ridge National Laboratory, Oak Ridge, Tennessee 37831, United States.

*Corresponding Authors: wangt@ornl.gov, dais@ornl.gov

KEYWORDS: *Direct recycling, Black mass, Ionic Liquid, Lithium battery recycling, Heavy Liquid Centrifugal Separation.*

Table S1: Metal composition of black mass determined by ICP-OES.

Element	Al	Co	Cu	Fe	Li	Mn	Ni
wt(%)	0.52	19.06	0.08	0.12	48.19	13.14	18.89

Material	Mass (g)	Yield (%)	Graphite (%)	NMC (%)
Black Mass	10	85	28	57

Table S2: Yield and composition of separated electrode active materials from black mass.

The total recovery rate (85%) was determined using the actual mass of black mass (10 g) introduced into the HLCS process. The sum of the recovered anode and cathode fractions

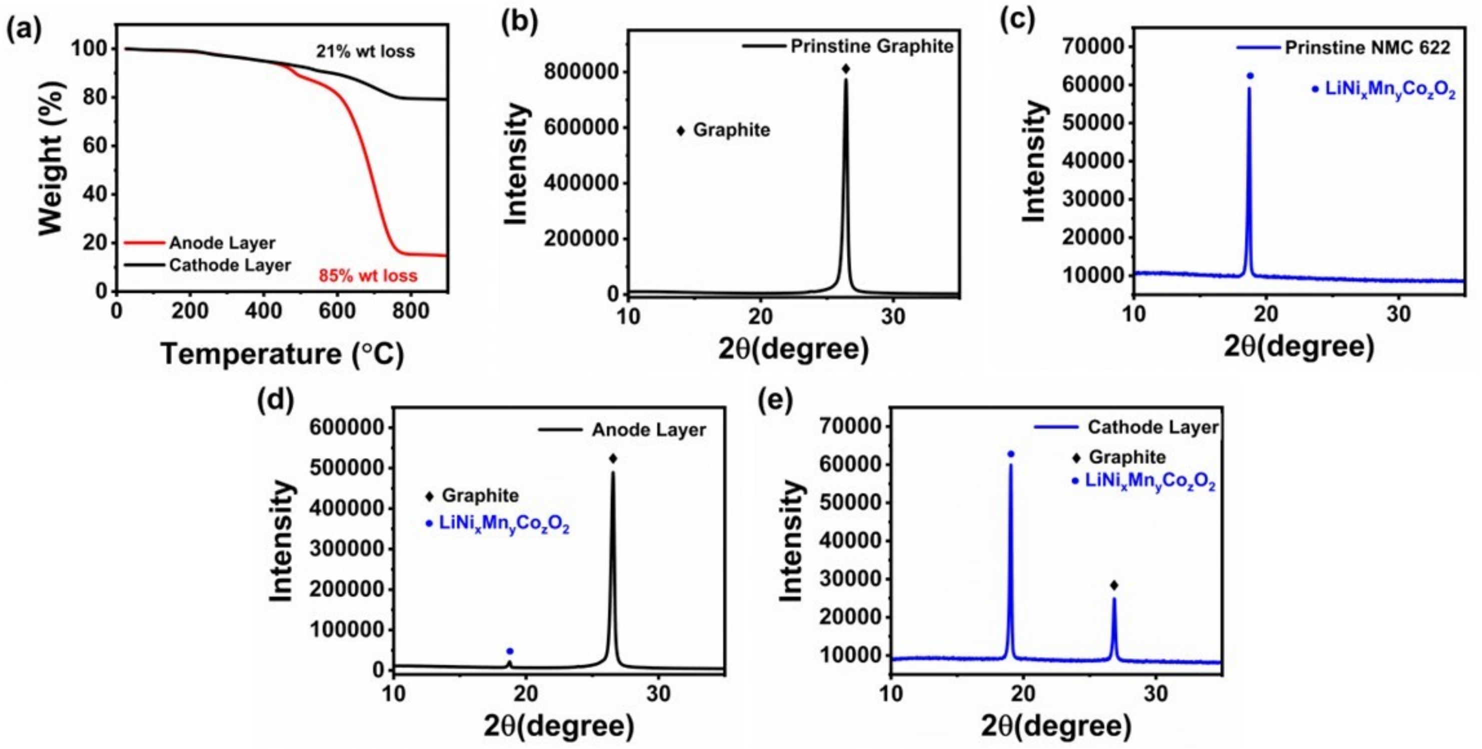

Figure S1: (a) TGA curves of the separated bottom and upper layers, XRD peaks of (b) pristine graphite (c) pristine NMC (d) anode layer and (d) cathode layer after separation

correspond to 8.5 g, equivalent to 85% of the total mass input.

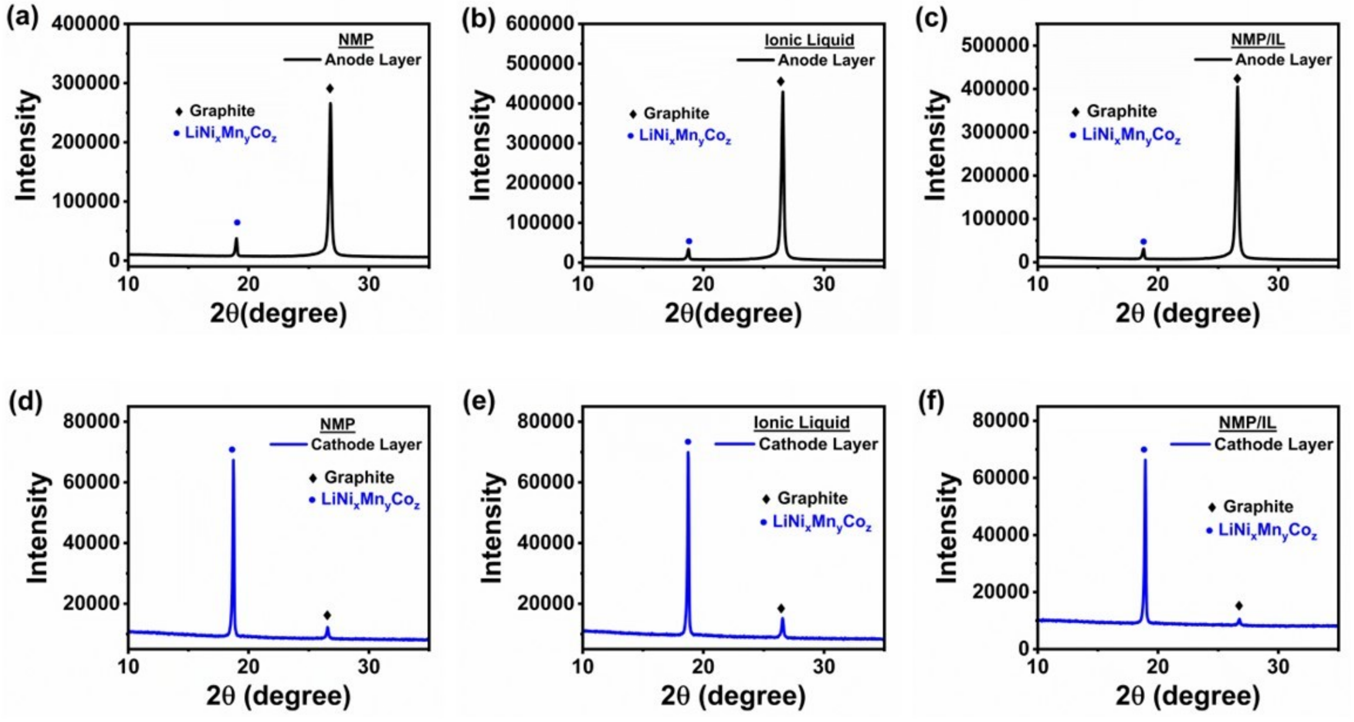

Figure S2: XRD pattern for different additives added to bromoform (a) NMP (b) Ionic Liquid (c) NMP/Ionic Liquid of the anode layer (d) NMP (e) Ionic Liquid (f) NMP/Ionic Liquid of the cathode layer

Table S3: The average crystal sizes of NMC calculated by Scherrer equation.

**Sample name**	**Average crystal size of NMC (nm)**
Pristine NMC 622	59.2
BF NMP IL UL	53.0
BF NMP IL BL	55.6
BF NMP UL	57.7
BF NMP BL	54.7
BF IL UL	55.9
BF IL BL	59.6

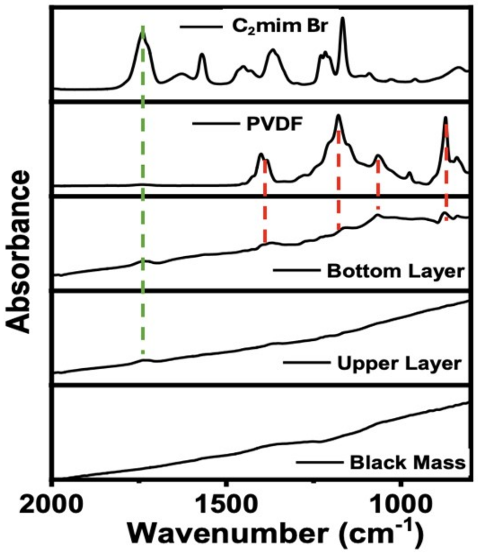

Figure S3. FTIR spectra of EMImBr, PVDF, black mass, and the products from the bottom and upper layers. The characteristic signals of EMImBr are marked with green dashed lines, and those of PVDF are marked with red dashed lines.

Due to the strong absorption of the black graphite and NMC powders, most functional-group signals are significantly attenuated, making peak identification difficult even in the black mass. Nonetheless, weak but identifiable peaks indicate that both the bottom- and upper-layer products contain characteristic signals from the IL, suggesting strong interactions between the IL and both NMC and graphite.

Although no clear PVDF peaks were observed in the FTIR spectra of the black mass or the upper-layer product, a series of PVDF-related peaks were detected in the bottom-layer product. This indicates partial removal of PVDF from graphite, which may contribute to breaking up particle agglomerates. Given the limited spectral information, it remains challenging to elucidate the detailed molecular mechanism solely from FTIR analysis.

However, the optical images provide more direct evidence of the IL's role in dispersing agglomerates. As shown in Figure S3, after stirring the black mass in either pure bromoform or a bromoform–IL mixture, a clear difference emerges: the bromoform–IL mixture forms a distinct interface between the upper and bottom layers, whereas pure bromoform does not. This visual contrast supports the conclusion that the ionic liquid effectively disrupts agglomeration.

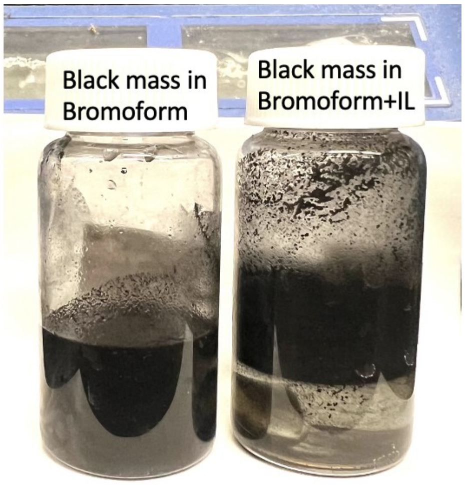

Figure S4. Optical images of black mass in pure bromoform and in a bromoform–IL mixture.

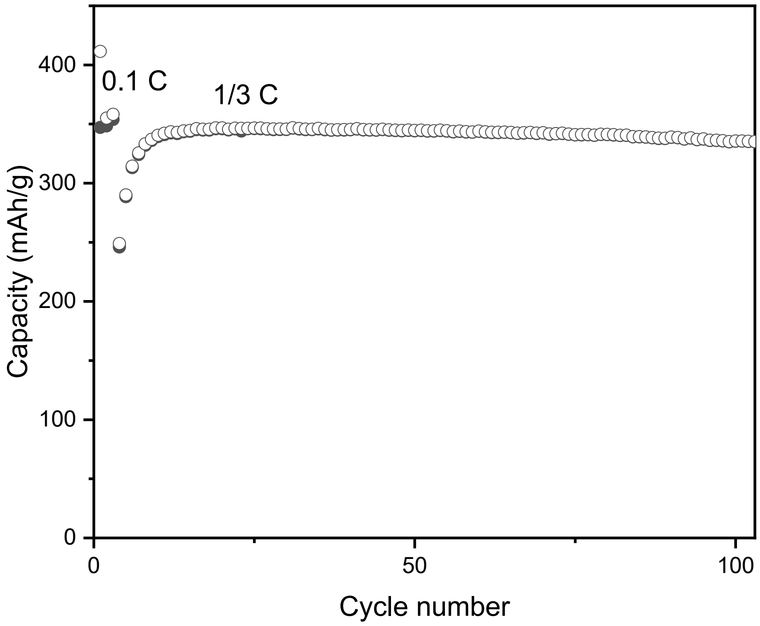

Figure S5: Half-cell cycling performance of anode layer after treatment.

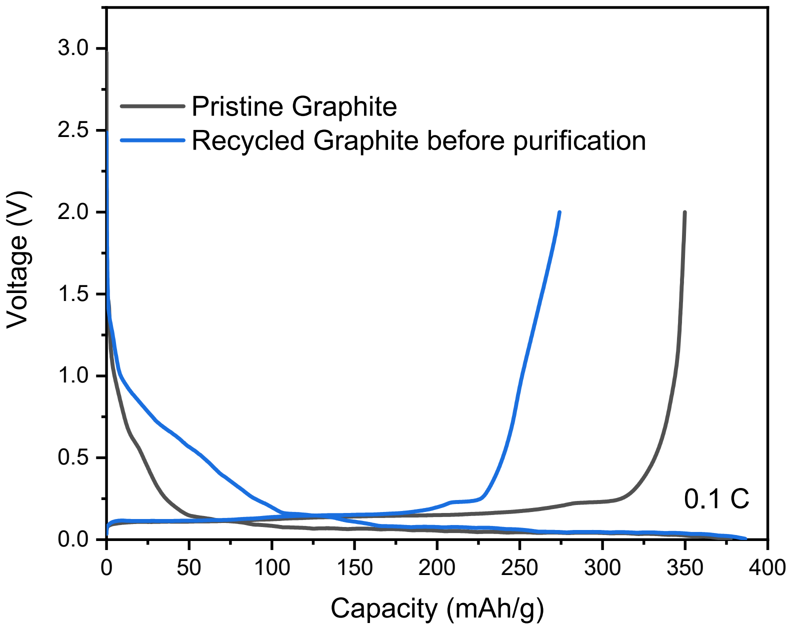

Figure S6: The first half-cell charge/discharge curves of pristine graphite and recovered graphite before purification. The purity of recovered graphite is 94 wt%.

The product before purification, with a purity of 94 wt%, exhibited a much lower initial coulombic efficiency than pristine graphite (71% vs. 92%, Figure S6).
